# Supplementary material for: A High-K+ Affinity Transporter (HKT) from Actinidia valvata Is Involved in Salt Tolerance in Kiwifruit
Source: Int J Mol Sci. 2023 Oct 30;24(21):15737. doi: 10.3390/ijms242115737 (PMC10647804; doi:10.3390/ijms242115737)
Supplement: Supplementary file 1 [file ijms-24-15737-s001.zip › ijms-2657223-supplementary.pdf]

Table S1. All the sequences of pair of primers used in the current study

All the sequences of pair of primers used in the current study			
Assay	Primer names	Primer Sequence	Restriction Site
Gene clon	AvHKT1(F)	ATGCCCAAGGACTTGGACTT	
	AvHKT1(R)	TCACATCATCACAACGAACACCA	
transformation	AvHKT1(F)	gagaacacgggggactctagaATGCCCAAGGACTTGGACTTG	*Xba*I,*Bam*H1
	AvHKT1(R)	gcccttgctcaccatggatccCATCATCACAACGAACACCACC	
transgenetic line detection	35S-30(F)	AACCTCCTCGGATTCCATTGCC	
	AvHKT(R)	TCACATCATCACAACGAACACCA	
ACTIN	F	TGCATGAGCGATCAAGTTTCAAG	
	R	TGTCCCATGTCTGGTTGATGACT	
qRT-PCR	YHKT（F）	CCTCGTGCCTACACCTTGTAA	
	YHKT（R）	TGGCTGAAACGCACGAAGAA	
subcelluar localization	pAN580-GFP-f	aagtccggagctagctctagatgcccaaggacttggacttgtttttcac	*Xba*I,*Bam*H1
	pAN580-GFP-r	agcggccgctgtacaggatccatcatcacaacgaacaccaccaatatagc	
Promoter clon	Promoter-F	TTCCCCATTACGCTGCTGAG	
	Promoter-R	CTTCCCCACCCACCAACATC	
